# Direct-Readout Optical Archival Storage in Fused Silica Using QR Microstructures

**DOI:** 10.3390/s26216721

**Published:** 2026-10-23

**Authors:** Jianping Li, Dongyang Guo, Wenjie Li, Wei Zhao

**Affiliations:** 1 Guangdong Provincial Key Lab of Robotics and Intelligent Systems, Shenzhen Institute of Advanced Technology, Chinese Academy of Sciences, Shenzhen 518055, China; jp.li2@siat.ac.cn; 2 University of Chinese Academy of Sciences, Beijing 101408, China; 3 School of Nano Science and Technology, University of Science and Technology of China, Suzhou 215127, China; 15034313473@163.com; 4 School of Computer Science and Artificial Intelligence, Shenzhen University of Advanced Technology, Shenzhen 518107, China; wj.li@siat.ac.cn

**Keywords:** optical storage, archival storage, Microscope image processing, two-photon lithography

## Abstract

Developing practical long-term optical archival storage technologies remains a significant challenge. Although high-density femtosecond-laser-based optical storage in fused silica offers exceptional durability, advanced volumetric approaches often require specialized readout systems, limiting practical deployment. Here, we propose a direct-readout optical storage framework based on surface-relief microstructures, combining two-photon lithography (TPL), reactive ion etching (RIE), and conventional microscopic imaging. Raw data are first encoded into QR codes and subsequently converted into three-dimensional QR microstructures for fabrication. A scan-line QR writing strategy is proposed, reducing the TPL writing time by approximately 80%. The fabricated QR microstructures are permanently transferred into fused silica by RIE and can be directly read using a conventional optical microscope. A hierarchy-aware QR reconstruction algorithm is further developed to recover complete QR codes from contour-dominated microscopic images. The proposed framework has an estimated geometric storage capacity of 2.85 GB per disc and a fabrication success rate of 94.5%, demonstrating a practical and highly accessible approach for long-term optical archival storage.

## 1. Introduction

The rapid growth of digital information driven by cloud computing, artificial intelligence (AI), high-performance computing (HPC), and the Internet of Things (IoT) has created an unprecedented demand for sustainable long-term archival storage. According to the International Data Corporation (IDC), the global data volume is expected to reach 394 ZB by 2028 [1]. While only a small fraction of these data remain actively accessed, the majority eventually becomes cold data that must be preserved for decades or even centuries with minimal maintenance, energy consumption, and migration cost [2]. Consequently, developing archival storage technologies that simultaneously provide extremely long lifetime, high density, low cost, and practical accessibility has become a major challenge in next-generation optical data storage [3,4].

Existing storage media are not well suited for long-term archival applications. Magnetic hard disk drives (HDDs) and solid-state drives (SSDs) typically provide operational lifetimes of only several decades and therefore require periodic data migration, which increases maintenance costs and introduces risks of data corruption and loss [5]. Although magnetic tape remains the dominant archival storage medium because of its low cost per terabyte, it also requires regular media refresh and suffers from slow sequential access. To overcome these limitations, a variety of emerging archival storage technologies have been actively investigated. DNA storage offers extraordinary storage density and theoretical preservation over thousands of years through synthetic nucleotide sequences [6,7]. Nitrogen-vacancy (NV) centers in diamond provide another attractive solution because of their exceptional chemical stability and optical addressability [8]. Although these technologies exhibit complementary advantages in archival storage, none simultaneously provides ultralong lifetime, practical accessibility, and low-cost readout.

Among the various emerging optical archival storage technologies, femtosecond-laser-written fused silica has attracted particular attention because of its exceptional thermal stability, radiation resistance, and theoretically extremely long storage lifetime [4,9,10]. Since the pioneering demonstration of three-dimensional optical data storage using nonlinear two-photon excitation [11], ultrafast laser processing has enabled increasingly sophisticated information encoding inside transparent materials. By exploiting femtosecond-laser-induced nanostructures [12,13], information can be encoded into multiple physical dimensions, including the spatial position, optical retardance, and orientation of self-assembled birefringent nanogratings. This concept has led to the development of five-dimensional (5D) optical storage, which has demonstrated ultrahigh storage density together with projected data lifetimes exceeding billions of years [14,15]. More recently, advances in laser writing technologies have continuously improved recording density and fabrication efficiency [16,17,18,19], while deep-learning-assisted decoding and error-correction strategies have significantly enhanced the reliability of information retrieval from laser-written nanostructures [20,21]. These technological advances have culminated in Microsoft’s Project Silica, which demonstrated cloud-scale archival storage using femtosecond-laser-written fused silica as a sustainable storage medium for future data centers [10].

In addition to birefringence-based multidimensional optical storage, direct optical readout based on laser-written binary dot patterns in fused silica has also been investigated. Watanabe et al. demonstrated a driveless read system in which femtosecond-laser-written binary dot patterns in fused silica were retrieved using a low-magnification optical microscope combined with image processing [22]. Shiozawa et al. subsequently extended this approach to simultaneous multi-bit and multilayer recording and demonstrated QR-code recording patterns that could be recovered from microscopic images [23]. These studies demonstrated that data stored in fused silica can be retrieved using conventional optical microscopy without polarization-sensitive imaging. Despite these advances, efficient fabrication and robust reconstruction remain important challenges for direct-readout glass storage.

To address these challenges, we propose a direct-readout optical storage framework based on surface-relief QR microstructures in fused silica, fabricated by two-photon lithography (TPL) and transferred by reactive ion etching (RIE). Unlike conventional birefringence-based storage systems, the proposed framework encodes information into geometric microstructures that can be imaged using conventional optical microscopy without polarization-sensitive readout. Different from previous volumetric laser-written direct-readout approaches, the present framework employs a surface-relief TPL–RIE fabrication route and specifically addresses fabrication efficiency and microscopic reconstruction. To improve fabrication efficiency, we further introduce a scan-line QR writing strategy that converts QR modules into a scan-line format compatible with the raster-scanning mechanism of TPL. A complete TPL–RIE fabrication workflow is established to transfer QR microstructures into fused silica, and QR microstructures with module widths down to 500 nm are successfully fabricated, demonstrating the high-resolution capability of the proposed fabrication process. Finally, a hierarchy-aware QR reconstruction algorithm is developed to reconstruct standard QR codes from contour-dominated microscopic images, enabling reliable decoding using only conventional optical microscopy.

## 2. Proposed Storage Framework

### 2.1. System Overview

**Figure 1:**
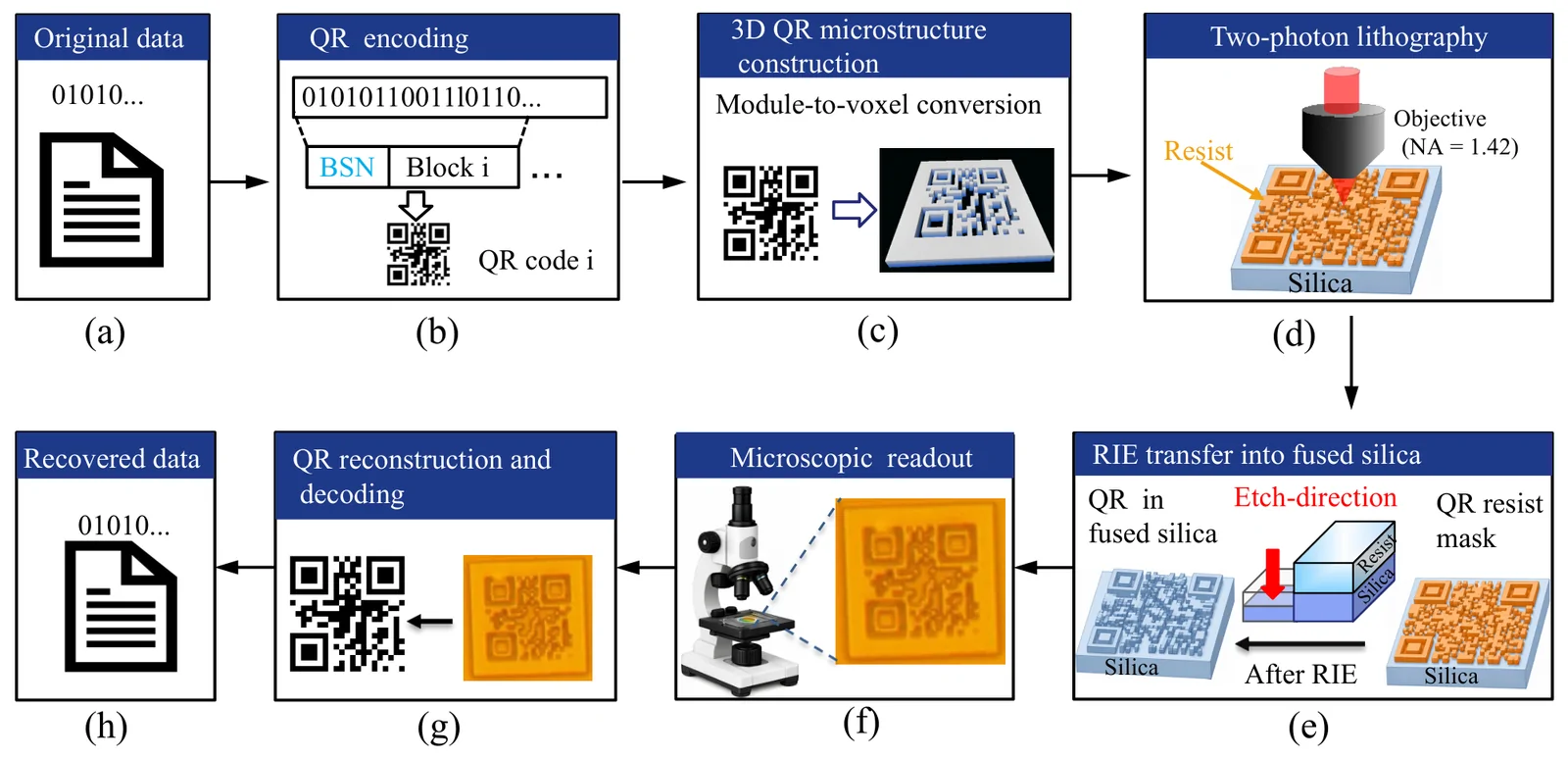
Workflow of the proposed archival data storage system. (**a**) Original digital data. (**b**) Quick Response (QR) encoding with Reed-Solomon error correction. (**c**) Conversion of QR modules into 3D microstructures. (**d**) Fabrication by two-photon lithography. (**e**) Transfer of QR microstructures into fused silica through reactive ion etching (RIE). (**f**) Microscopic readout of the stored QR structures. (**g**) QR reconstruction and decoding. (**h**) Recovery of the original data.

The proposed long-term data storage system is illustrated in Figure 1. The original data are received as a stream of bits (Figure 1a). The input bitstream is divided into multiple data blocks. Each block is prefixed with a Block Sequence Number (BSN) to form a data packet, which is then encoded into a Quick Response (QR) code with embedded Reed-Solomon (RS) error correction (Figure 1b).

Each QR code is then converted into a three-dimensional QR microstructure through a module-to-voxel mapping strategy, where each QR module is represented by a predefined voxel unit suitable for fabrication (Figure 1c). The designed QR microstructure is fabricated in a photoresist-coated fused silica substrate using two-photon lithography (TPL) (Figure 1d). After development, the patterned photoresist serves as an etching mask, and the QR microstructure is permanently transferred into the fused silica substrate through reactive ion etching (RIE), resulting in a permanent storage medium (Figure 1e). The stored QR microstructure is then read using an optical microscope, and the acquired microscopic image is used for subsequent processing (Figure 1f). The captured microscopic image is first enhanced and then processed using the proposed hierarchy-aware contour filling algorithm to reconstruct a standard QR code, which is subsequently decoded to recover the encoded data packets (Figure 1g). Finally, the recovered data packets are reordered according to their Block Sequence Numbers (BSNs) and concatenated to reconstruct the original bitstream, thereby achieving reliable long-term data retrieval (Figure 1h).

### 2.2. QR Encoding and 3D Microstructure Conversion

Before physical fabrication, the input data must be converted into a representation that is suitable for both long-term storage and reliable retrieval. The encoding scheme should satisfy two essential requirements. First, the encoded data should be represented in a two-dimensional format that can be readily transformed into printable microstructures for two-photon lithography. Second, the encoding should provide sufficient error resilience to ensure reliable data recovery in the presence of fabrication imperfections, material degradation, or imaging noise.

To meet these requirements, the proposed system adopts the Quick Response (QR) code as the fundamental storage unit. Compared with conventional binary encoding, QR codes incorporate standardized Reed-Solomon (RS) error correction, enabling robust data recovery even when part of the stored pattern is damaged or degraded. In addition, their regular matrix structure is naturally suited for conversion into three-dimensional microstructures.

As illustrated in Figure 1b, the input bitstream is first partitioned into multiple data blocks according to the storage capacity of the selected QR code version. A Block Sequence Number (BSN) is then prefixed to each block to form a data packet, allowing all decoded packets to be reordered during data reconstruction. Each packet is subsequently encoded into an individual QR code using the standard QR encoding with embedded Reed-Solomon (RS) error correction.

**Figure 2:**
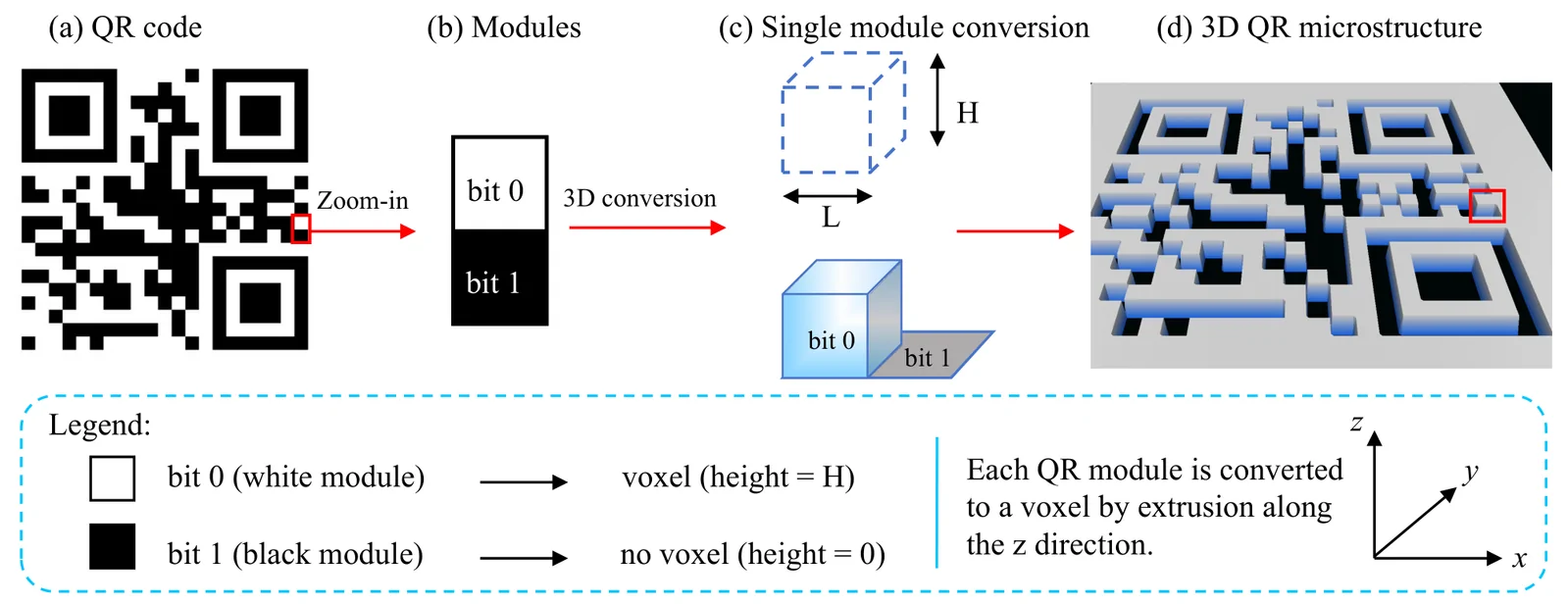
QR-code-to-3D microstructure conversion. (**a**) QR code. (**b**) Zoom-in shows the binary modules (0:white, 1:black). (**c**) Each module is converted to a voxel by extrusion with lateral size L and height H. (**d**) Complete 3D QR microstructure.

The generated QR code is subsequently converted into a three-dimensional microstructure for TPL fabrication. As shown in Figure 2, each white module is represented by a cuboid voxel with a predefined lateral dimension L and height H, while black modules remain unexposed. This one-to-one mapping preserves the topology of the original QR code while providing a fabrication-ready three-dimensional model. In this work, each QR module is represented by a designed cuboid microstructure unit with lateral dimension (L) and height (H), as illustrated in Figure 2. This geometric storage unit is distinct from the TPL polymerization voxel, which refers to the localized volume polymerized near the laser focal region. The binary information is physically encoded by two surface-height states after RIE transfer.

### 2.3. Scan-Line QR Writing Strategy for Efficient TPL Fabrication

**Figure 3:**
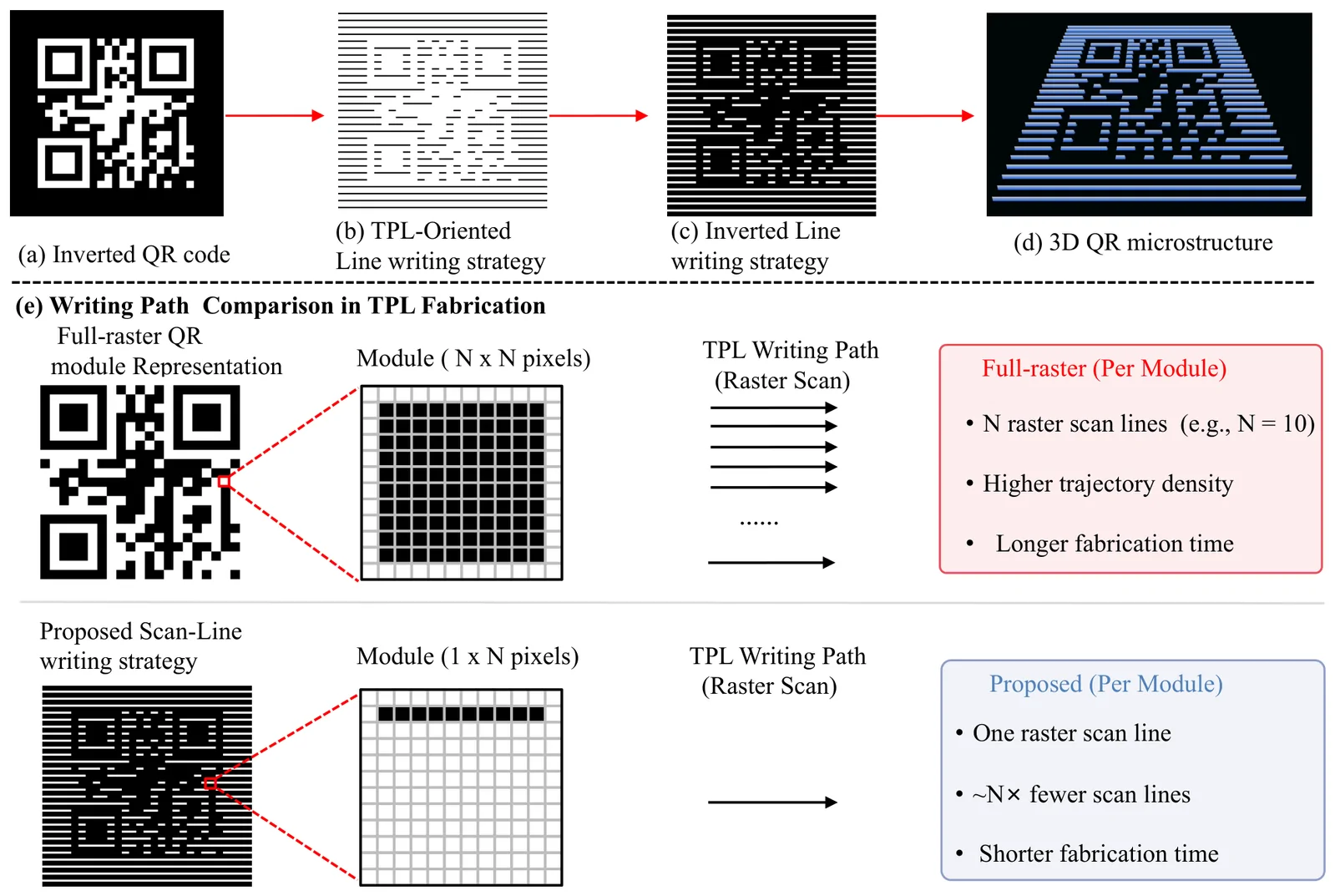
Transformation from a full-raster QR module representation to the proposed scan-line QR writing strategy. (**a**) Conventional inverted QR code. (**b**) Proposed scan-line QR writing strategy. (**c**) Inverted proposed scan-line writing strategy. (**d**) 3D visualization of the proposed scan-line QR writing strategy. (**e**) Comparison of the raster scan trajectories required for TPL fabrication, illustrating the reduction in scan lines achieved by the proposed writing strategy.

Full-raster QR module representations are composed of square modules, each of which is typically represented by multiple raster scan lines during two-photon lithography (TPL), resulting in redundant scanning trajectories and increased fabrication time. To reduce the writing workload while preserving the encoded information, we propose a scan-line QR writing strategy, as illustrated in Figure 3. Instead of exposing every pixel within a QR module, only one representative raster scan line is retained for each module. Consequently, the number of raster scan lines required for fabrication is reduced by approximately a factor of N (where N is the module height in pixels), thereby significantly shortening the writing process. During data retrieval, the omitted pixels are reconstructed by image processing before QR decoding, enabling lossless recovery of the encoded information.

**Fabrication-Oriented Design Principle.** The proposed scan-line writing strategy is designed to optimize the fabrication process rather than modify the encoded information. Specifically, only the geometric representation of each QR module is simplified for TPL writing, while the logical information carried by the QR code remains unchanged. During readout, the standard QR geometry is restored through image processing prior to decoding, ensuring lossless recovery of the original data. It should be emphasized that the proposed method does not alter the QR encoding scheme itself; instead, it introduces a fabrication-friendly geometric representation that minimizes the TPL writing workload while preserving full compatibility with standard QR decoding after reconstruction.

### 2.4. Two-Photon Lithography and Etching Transfer

The designed QR microstructures were fabricated and permanently transferred into fused silica through two successive processes, namely two-photon lithography (TPL) for photoresist patterning and reactive ion etching (RIE) for pattern transfer, as illustrated in Figure 1d,e. Two-photon lithography (TPL) is a maskless micro/nanofabrication technique capable of directly fabricating arbitrary three-dimensional microstructures with submicrometer resolution by exploiting nonlinear two-photon polymerization [24,25,26]. Owing to its exceptional design flexibility, high fabrication accuracy, and true three-dimensional writing capability, TPL has become one of the most powerful techniques for fabricating complex micro/nanostructures in optics, photonics, and microdevices [27,28,29,30,31], making it particularly suitable for fabricating the proposed QR microstructures.

The proposed storage architecture is intended for long-term archival applications following the Write Once, Read Many (WORM) paradigm, where data are recorded once and retrieved repeatedly over extended periods. Although single-photon lithography may offer higher throughput for two-dimensional patterning, TPL was selected for its high spatial resolution, maskless fabrication capability, and flexible control of microstructure geometry. In this work, a commercial TPL system (Nanolitho 2PP, YuZhiQuan Instruments, Hangzhou, China) equipped with a femtosecond laser (515 nm wavelength, 200 fs pulse duration, and a repetition rate of 80 MHz) was employed to fabricate the designed QR microstructures in a negative-tone photoresist coated on fused silica substrates. The system employs a bimolecular photosensitizer-photoacid generator (PS-PAG) mechanism, enabling high-speed fabrication (up to 100 mm/s) with sub-200 nm resolution [32]. A high-numerical-aperture objective lens (NA = 1.42) was used to tightly focus the laser beam. The fabrication parameters were experimentally optimized, and the optimized conditions are presented in Section 3.1.

Following TPL fabrication, the patterned photoresist served as an etching mask for transferring the QR microstructures into the fused silica substrate by reactive ion etching (RIE). Fluorine-based RIE is widely used for transferring high-resolution photoresist patterns into fused silica because it provides highly anisotropic etching with excellent pattern fidelity [33,34]. Accordingly, a CF_4_/Ar plasma was employed for pattern transfer. The etching parameters were experimentally selected, as described in Section 3.4. After etching, ultrasonic cleaning was performed to remove the residual photoresist and expose the transferred fused silica microstructures for subsequent characterization.

### 2.5. Microscopic Readout and QR Code Reconstruction

Under certain illumination conditions, microscopic images of conventionally fabricated QR microstructures often exhibit contour-dominated appearances, in which only the module boundaries remain clearly visible while the interior regions become nearly indistinguishable from the background owing to optical scattering and limited intensity contrast. Consequently, conventional image enhancement and binarization methods cannot reliably reconstruct complete QR modules.

**Figure 4:**
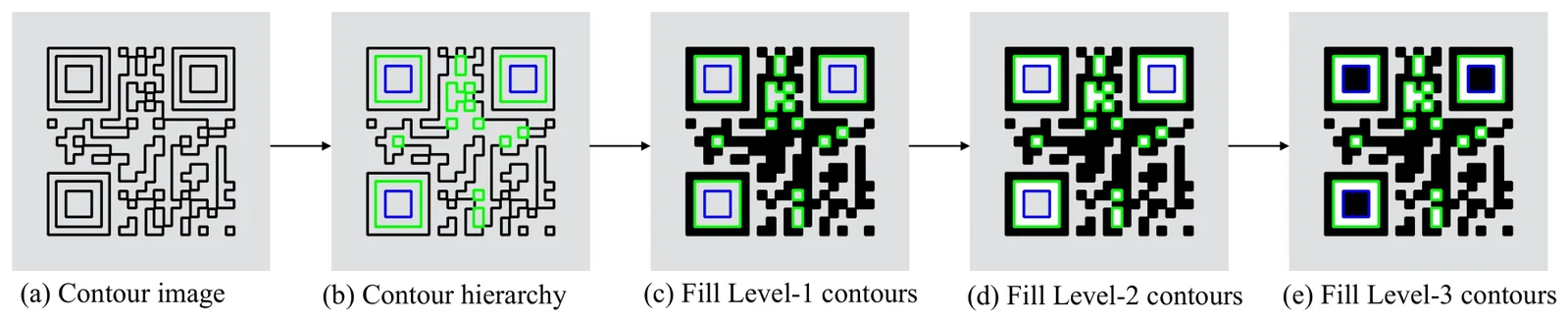
Illustration of the proposed hierarchy-aware contour filling algorithm. (**a**) Captured contour image. (**b**) Parent–child contour hierarchy obtained by contour analysis. (**c**) Filling Level-1 contours with black. (**d**) Filling Level-2 contours with white. (**e**) Filling Level-3 contours with black, resulting in the reconstructed QR code.

To overcome this limitation, a hierarchy-aware contour filling algorithm is proposed to reconstruct the QR code from its preserved contour topology prior to decoding, as illustrated in Figure 4. The captured microscopic images are first restored using a deblurring algorithm to compensate for optical degradation, followed by Gaussian filtering to suppress residual imaging noise. Background compensation is then applied to reduce illumination non-uniformity, and adaptive thresholding is subsequently performed to generate a binary contour image suitable for contour extraction. After contour extraction, all contours are organized into a parent–child hierarchy according to their nesting relationships, as shown in Figure 4b. Starting from each outermost contour, the enclosed region is filled with black, while successive child contours are recursively filled with alternating colors (white and black), as illustrated in Figure 4c–e. This recursive filling procedure exploits the hierarchical nesting relationships among contours rather than relying on grayscale intensity information, thereby restoring the alternating black–white topology of the QR code while preserving its structural relationships. The proposed reconstruction algorithm effectively compensates for illumination-induced contour-only imaging, enabling complete QR recovery and reliable decoding using conventional optical microscopy under varying illumination conditions.

## 3. Results and Discussion

### 3.1. Optimization of TPL Fabrication Parameters

**Figure 5:**
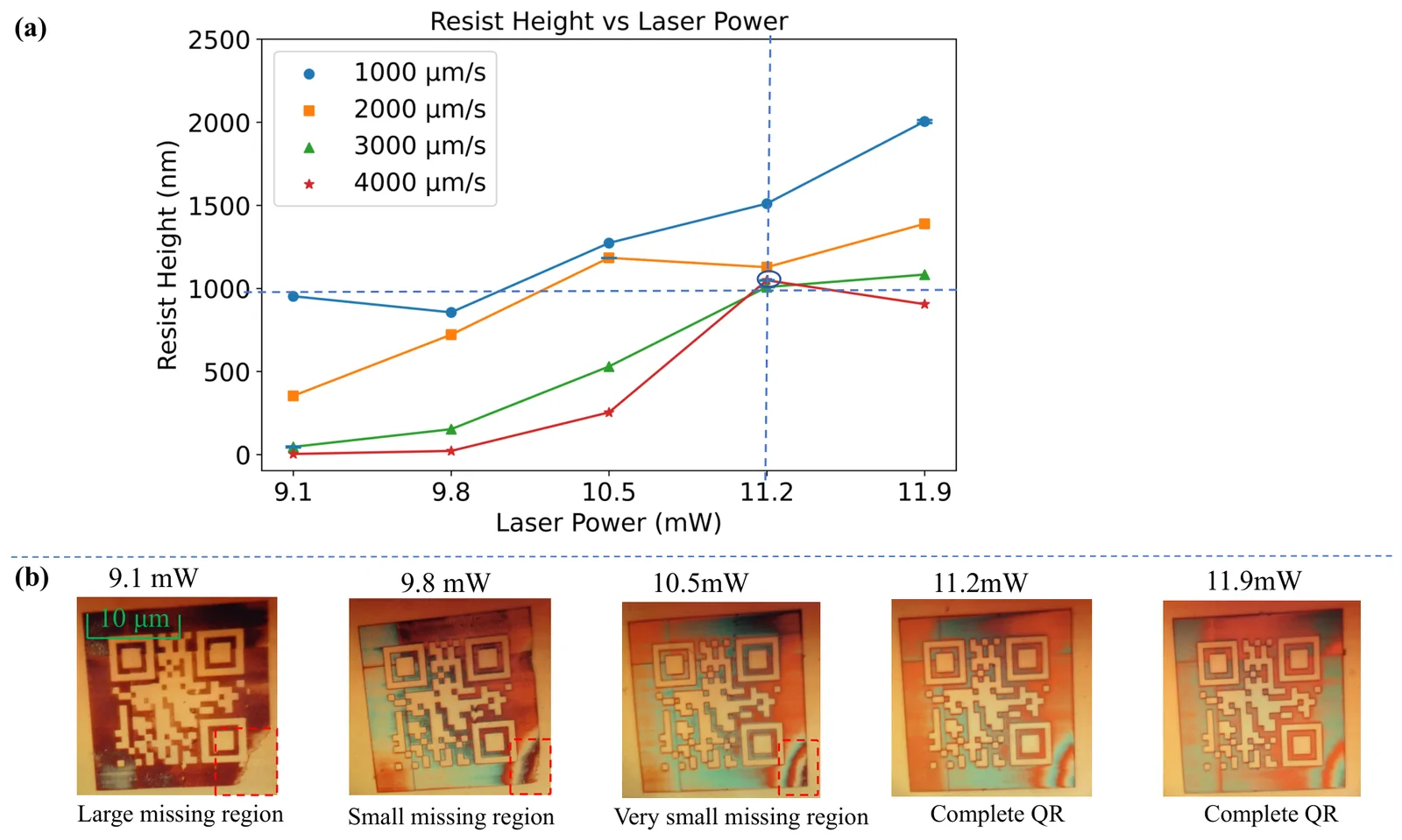
Optimization of two-photon lithography (TPL) parameters for QR microstructure fabrication. (**a**) Fabricated photoresist height versus laser power at different scanning speeds. Error bars at five representative conditions indicate the standard deviation of measurements from three independently fabricated structures (n = 3). These error bars represent structure-to-structure variation rather than variation between separate fabrication batches. Some error bars are smaller than the data markers. (**b**) Optical microscope images of QR microstructures fabricated at a scanning speed of 1000 μm/s with laser powers of 9.1, 9.8, 10.5, 11.2, and 11.9 mW.

To optimize the fabrication parameters for QR microstructures, a series of experiments were carried out using the two-photon lithography (TPL) system with a fixed maximum laser power of 35 mW. The laser power was varied from 9.1 to 11.9 mW (26–34% of the 35 mW reference power) in steps of 0.7 mW, while the scanning speed was set to 1000, 2000, 3000, and 4000 μm/s, respectively. The designed photoresist height was 1μm, and the module width was 1μm. After fabrication, the photoresist height was measured using a surface profilometer, and the results are summarized in Figure 5a, where the x-axis denotes “Laser Power (% of 35 mW)”.

As shown in Figure 5a, the fabricated photoresist height generally increases with increasing laser power, whereas increasing the scanning speed tends to reduce the fabricated height because of the reduced exposure per unit length. At low laser powers (9.1–9.8 mW), the photoresist height decreases rapidly with increasing scanning speed, indicating insufficient exposure for complete polymerization. When the laser power reaches 11.2 mW, a photoresist height of approximately 1 μm is obtained at a scanning speed of 3000 μm/s, which closely agrees with the designed structure height. The selection of the writing parameters was primarily based on the deviation of the fabricated resist height from the designed height of 1 μm, together with writing efficiency and structural completenessm, together with writing efficiency and structural completeness. At 11.2 mW laser power and 3000 μm/s scanning speed, the measured resist height was 1007.8 ± 1.8 nm (mean ± SD, n=3), corresponding to a relative height deviation of approximately 0.78%. This condition provides a relatively high scanning speed while maintaining the fabricated height close to the designed value. Therefore, 11.2 mW laser power and 3000 μm/s were selected as the operating parameters for subsequent experiments.

To assess the reproducibility of the resist-height measurements, additional measurements were performed on three independently fabricated structures (n=3) at five representative power–speed conditions spanning the investigated parameter range, including the selected fabrication condition. The results are reported as the mean ± standard deviation in Figure 5a. The measured standard deviations ranged from 0.6 to 9.1 nm, corresponding to relative standard deviations below 2% for all five conditions. At the selected condition of 11.2 mW laser power and 3000 μm/s scanning speed, the resist height was 1007.8±1.8 nm (mean ± SD, n=3).

To further evaluate the influence of laser power on QR fabrication fidelity, QR microstructures were fabricated at a fixed scanning speed of 1000 μm/s using laser powers ranging from 9.1 mW to 11.9 mW, and the resulting patterns were examined under an optical microscope, as shown in Figure 5b. At 26%, severe defects are observed in the lower-right region of the QR code, indicating incomplete polymerization. Increasing the laser power to 9.8 mW significantly reduces the missing region, although local defects remain visible. At 10.5 mW, only minor discontinuities are observed. Complete QR microstructures are obtained at 11.2 mW, and no noticeable improvement is observed when the laser power is further increased to 11.9mW. These observations are consistent with the photoresist height measurements shown in Figure 5a, confirming that 11.2 mW laser power provides sufficient exposure to achieve complete QR fabrication while avoiding unnecessary overexposure.

### 3.2. Fabrication Efficiency of the Scan-Line Writing Strategy

To evaluate the effectiveness of the proposed scan-line QR writing strategy in improving fabrication efficiency, the TPL writing time was compared with that of the full-raster module representation for QR Versions 1, 6, and 9. These versions contain 21×21, 41×41, and 53×53 modules, respectively. With a designed module width of 1 μm, the corresponding nominal QR pattern dimensions are 21×21, 41×41, and 53×53 μm^2^, excluding quiet zones. Both writing strategies were evaluated using the same encoded QR patterns and identical fabrication parameters, including a laser power of 11.2 mW, a scanning speed of 3000 μm/s, and a designed module width of 1 μm. Therefore, the two strategies represent the same logical information within the same nominal QR pattern area, while differing in their exposure trajectories.

**Figure 6:**
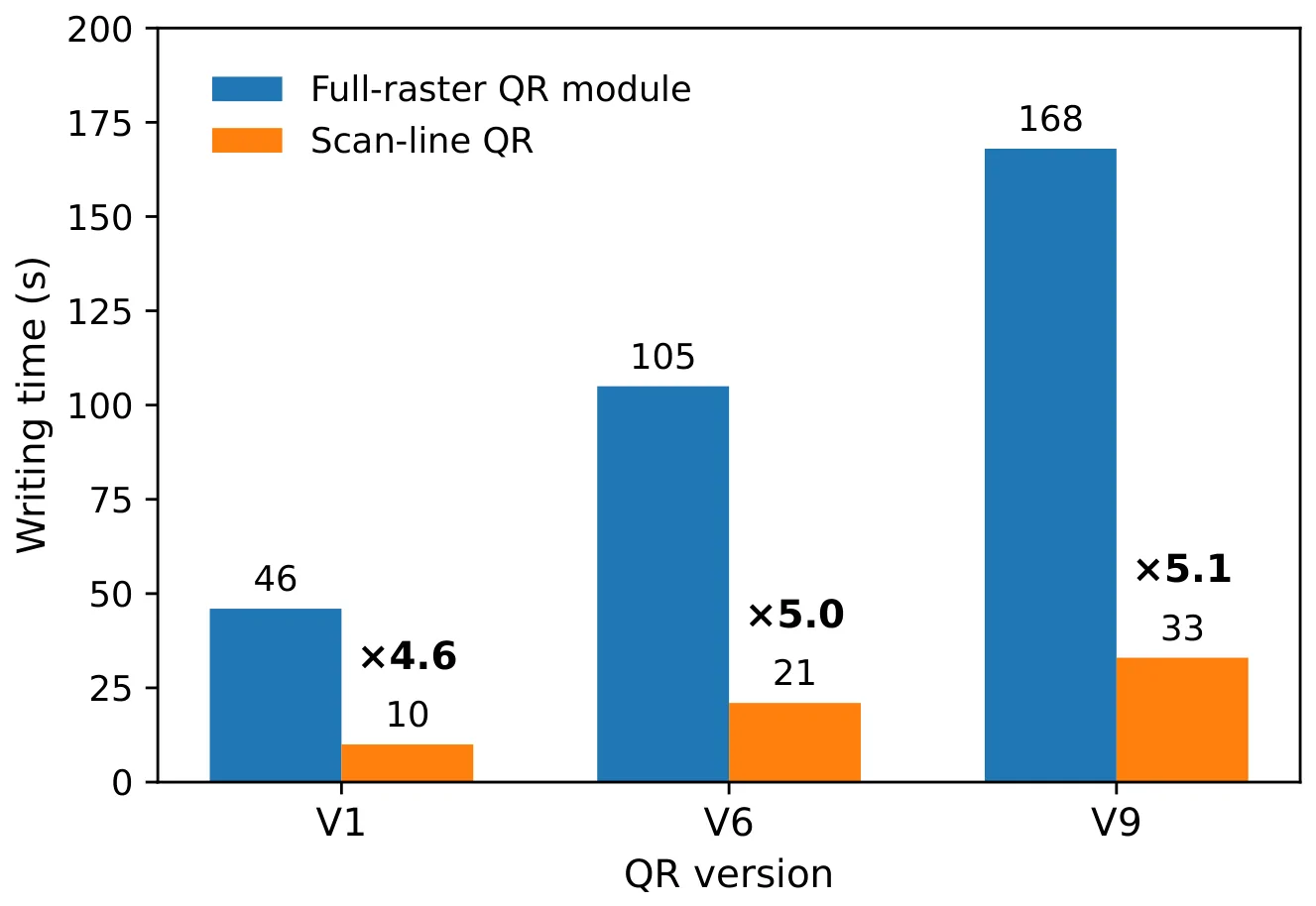
Comparison of TPL writing time between the full-raster module representation and the proposed scan-line QR writing strategy for QR Versions 1, 6, and 9.

Figure 6 shows that the proposed scan-line writing strategy consistently requires significantly shorter writing times than the full-raster module representation for all tested QR versions. Specifically, the writing time is reduced from 46 s to 10 s for Version 1, from 105 s to 21 s for Version 6, and from 168 s to 33 s for Version 9, corresponding to speedup factors ranging from 4.6× to 5.1×. The improvement originates from the proposed scan-line QR writing strategy, which is specifically designed to match the raster-scanning mechanism of TPL. In the full-raster module representation, each QR module is fabricated using multiple raster scan lines corresponding to the module height. In contrast, the proposed scan-line writing strategy retains only a single representative scan line for each module while preserving the encoded information. As a result, the total number of raster scan lines required for fabrication is significantly reduced, leading to a substantial decrease in the total writing time. These results demonstrate that the proposed scan-line writing strategy significantly improves TPL fabrication efficiency, providing an effective strategy for fabricating QR microstructures for long-term optical storage.

### 3.3. Fabrication Reliability of QR Microstructures

**Figure 7:**
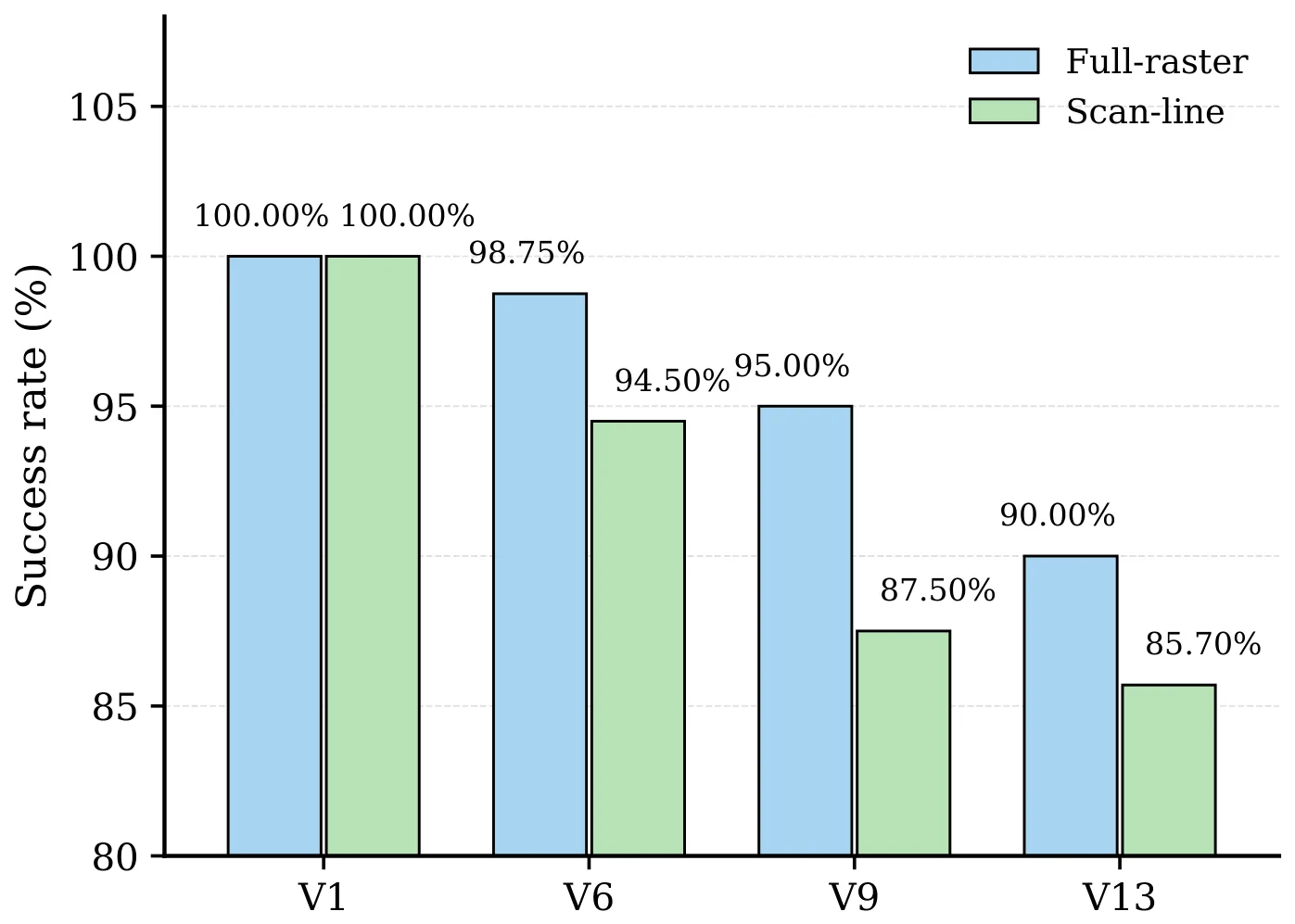
Comparison of fabrication success rates for QR microstructures of different versions using full-raster (light blue) and scan-line (light green) writing strategies. Both strategies were evaluated under identical fabrication conditions, with designed module dimensions of 1μm ×1μm ×1μm. Fabrication success was defined as the absence of missing or detached QR modules.

To quantitatively compare the fabrication reliability of the two writing strategies, QR microstructures of Versions 1, 6, 9, and 13 were fabricated using both full-raster and scan-line writing. Both strategies were evaluated under identical fabrication conditions, including a laser power of 11.2 mW, a scanning speed of 3000 μm/s, and designed module dimensions of 1μm ×1μm ×1μm. Fabrication success was defined as the absence of missing or detached QR modules. The success rates of the two strategies were calculated separately to enable a direct comparison, as shown in Figure 7. The fabrication success rate was defined as the number of successfully fabricated QR microstructures divided by the total number evaluated for the corresponding writing strategy and QR version. The reported success rates represent structure-level proportions rather than batch-to-batch variability.

As shown in Figure 7, the fabrication success rates of full-raster writing were 100%, 98.75%, 95.0%, and 90.0% for QR Versions 1, 6, 9, and 13, respectively. The corresponding success rates of scan-line writing were 100%, 94.5%, 87.5%, and 85.7%. Both strategies exhibited decreasing fabrication success rates with increasing QR version, while scan-line writing showed slightly lower reliability for the higher versions. Since the designed module dimensions remained constant, this trend may be associated with the increasing structural complexity and the greater number of isolated features in higher-version QR patterns, which are more susceptible to detachment during fabrication and subsequent processing.

For QR Version 6, the fabrication success rate of scan-line writing was 94.5%, compared with 98.75% for full-raster writing, corresponding to a difference of 4.25 percentage points. Nevertheless, scan-line writing reduced the fabrication time from 105 to 21 s, representing an 80% reduction. These results indicate that the proposed scan-line strategy substantially improves writing efficiency at the cost of a moderate reduction in fabrication success rate. Therefore, QR Version 6 was selected as a practical compromise between data capacity, fabrication efficiency, and structural reliability for subsequent experiments.

The QR code version determines the number of modules in the QR matrix, with Version *V* containing (4V+17)×(4V+17) modules. Accordingly, Versions 1, 6, 9, and 13 consist of 21 × 21, 41 × 41, 53 × 53, and 69 × 69 modules, respectively. Higher versions provide greater data capacity but also introduce more complex module arrangements, potentially increasing the number of isolated features susceptible to fabrication defects. Because the designed QR module dimensions are kept constant for all QR versions, the decrease in fabrication success rate cannot be attributed to reduced feature size. Instead, higher-version QR codes contain a substantially larger number of isolated modules, which possess weaker mechanical support during photoresist development and subsequent reactive ion etching. Consequently, these isolated modules are more susceptible to detachment, leading to a gradual decrease in fabrication success rate.

**Figure 8:**
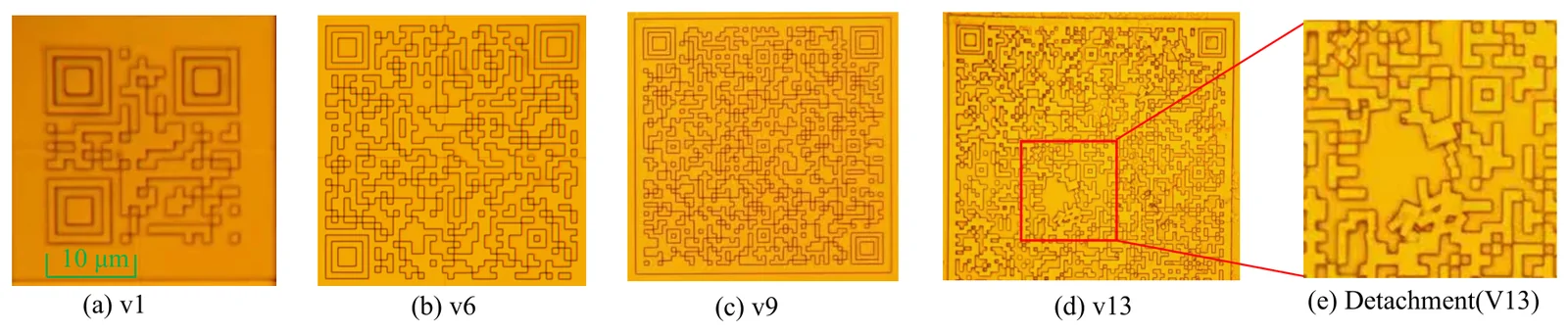
Representative optical microscope images of fabricated QR microstructures. (**a**) Version 1. (**b**) Version 6. (**c**) Version 9. (**d**) Version 13. (**e**) Enlarged view of the detached module highlighted in (**d**), illustrating the dominant fabrication failure mode observed in higher-version QR codes.

Representative optical microscope images are shown in Figure 8. Versions 1, 6, and 9 exhibit complete QR codes without noticeable defects, whereas isolated module detachment is observed in Version 13, as highlighted in Figure 8e. This representative failure mode agrees well with the statistical results in Figure 7, confirming that the gradual reduction in fabrication reliability originates primarily from the increasing number of isolated modules in higher-version QR codes rather than from the fabrication resolution itself.

Considering both storage capacity and fabrication reliability, Version 6 was selected as the practical version for experiments. Compared with Version 1, Version 6 provides substantially higher storage capacity while maintaining a high fabrication success rate of 94.5%. Although higher QR versions further increase storage capacity, the accompanying increase in isolated modules leads to a higher probability of structural detachment during fabrication. Therefore, Version 6 was selected for the subsequent RIE-transfer and capacity-estimation experiments.

### 3.4. Transfer of QR Microstructures into Fused Silica

To transfer the fabricated QR microstructures into fused silica, the RIE process parameters were selected primarily based on the achievable etching depth. The objective was to obtain a sufficient surface-relief depth while preserving the QR microstructure geometry. The selected parameters are summarized in Table 1.

**Figure 9:**
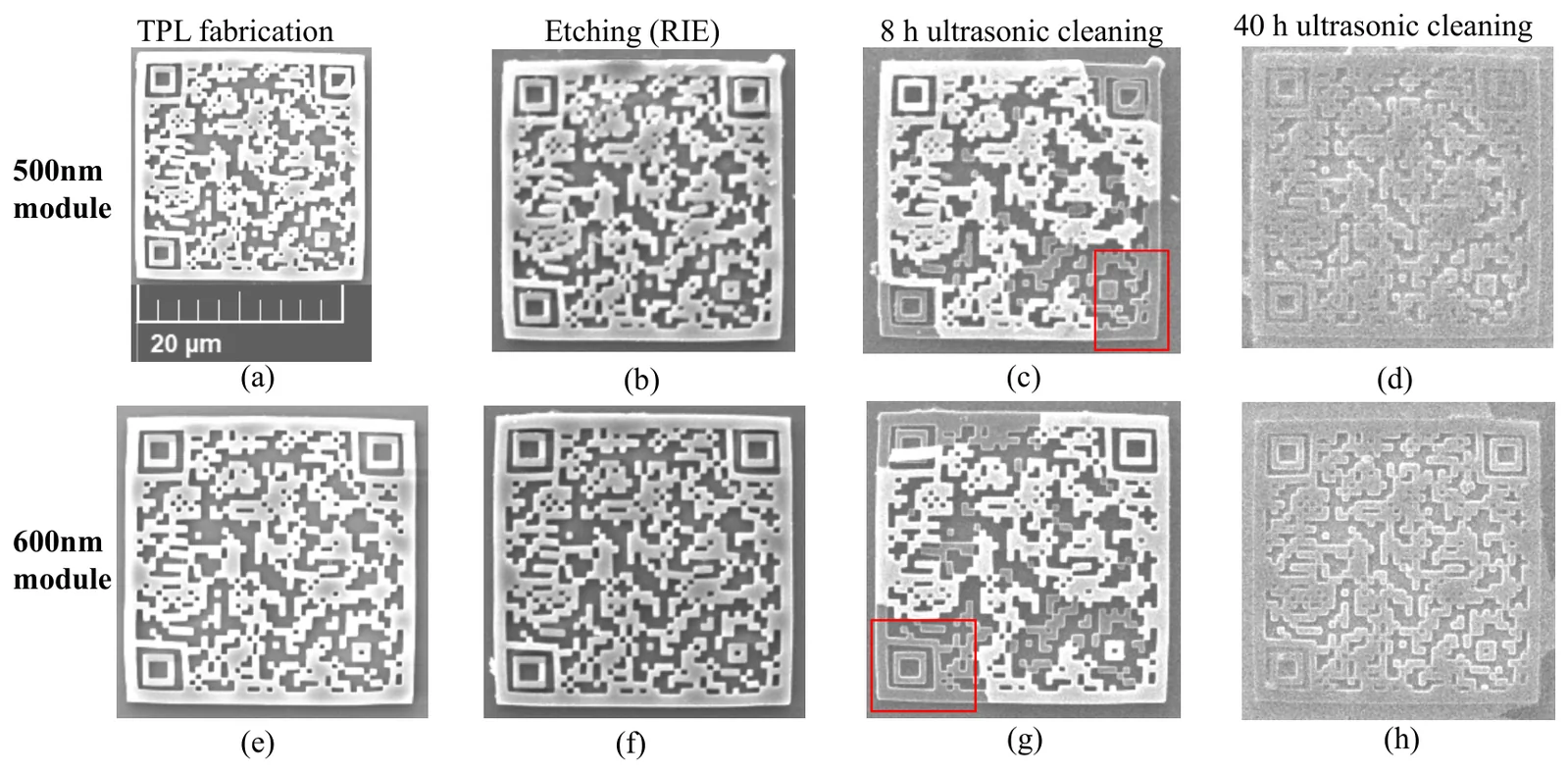
SEM images showing the morphological evolution of QR microstructures during the proposed TPL–RIE fabrication process. The top row corresponds to QR microstructures with a module width of 500 nm, while the bottom row corresponds to a module width of 600 nm. The columns represent successive fabrication stages: (**a**,**e**) after TPL fabrication, (**b**,**f**) after RIE transfer, (**c**,**g**) after 8 h ultrasonic cleaning, and (**d**,**h**) after 40 h ultrasonic cleaning.

To evaluate the effectiveness of the optimized TPL–RIE fabrication strategy, QR microstructures with module widths of 500 nm and 600 nm were fabricated and characterized at different fabrication stages. The corresponding SEM images are presented in Figure 9, where each row corresponds to a different module size and each column represents a successive fabrication stage, namely TPL fabrication, RIE transfer, 8 h ultrasonic cleaning, and 40 h ultrasonic cleaning.

As shown in Figure 9a,e, both module sizes produce well-defined QR microstructures with clear boundaries immediately after TPL fabrication, indicating that the selected writing parameters enable stable fabrication of the designed microstructures. After reactive ion etching (Figure 9b,f), the overall QR geometry is preserved, demonstrating successful pattern transfer from the photoresist mask into the fused silica substrate.

**Figure 10:**
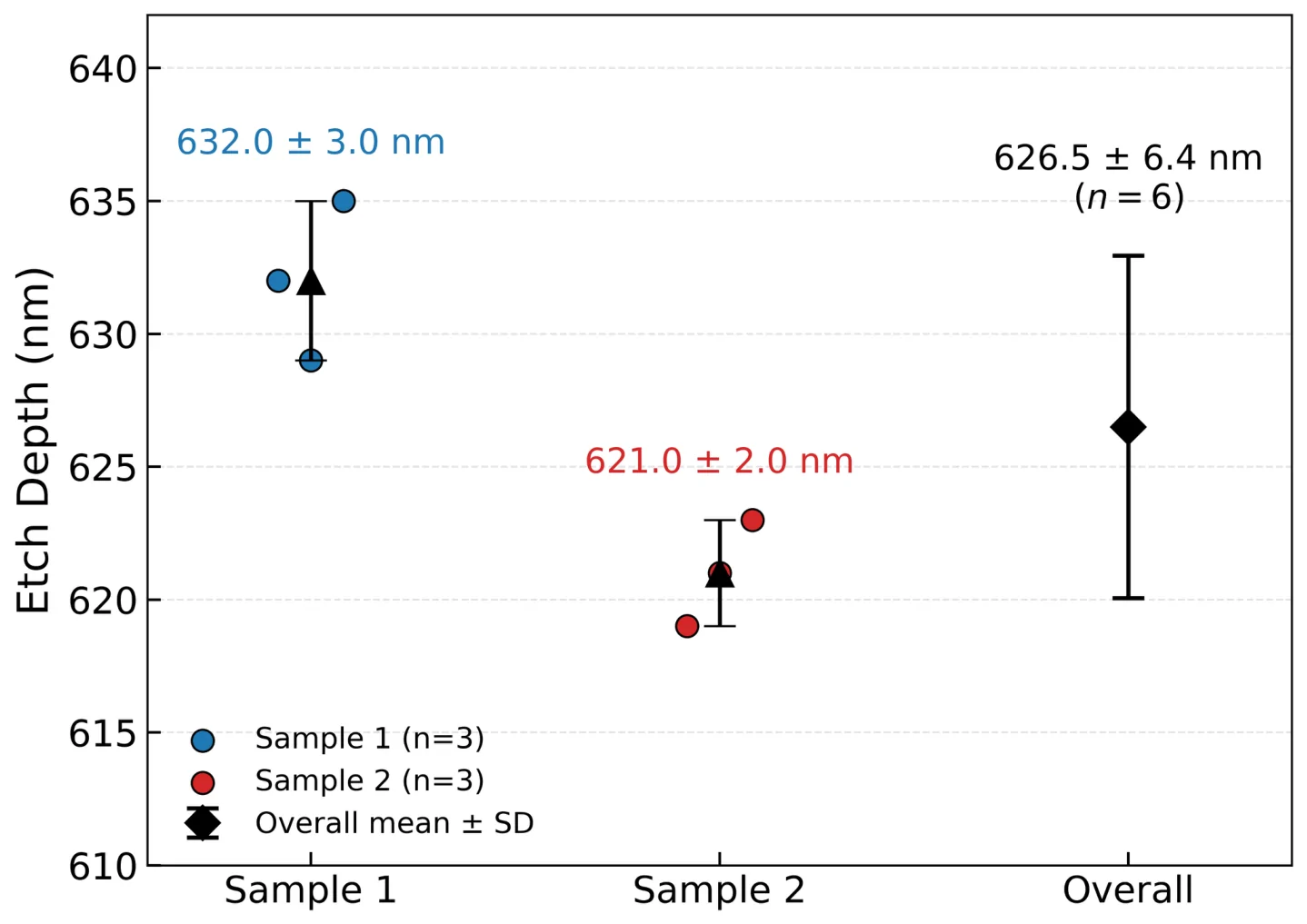
Stylus-profilometer measurements of the RIE-transferred etch depth. Individual measurements from three locations on each of two independently fabricated samples are shown together with the overall mean ± SD (626.5 ± 6.4 nm, *n* = 6).

Because the photoresist mask remains after RIE, ultrasonic cleaning was performed to gradually remove the residual photoresist and expose the transferred fused silica microstructures. After 8 h of ultrasonic cleaning, portions of photoresist were removed, revealing the transferred fused silica microstructures, as shown in Figure 9c,g. The coexistence of the exposed fused silica structures and the remaining photoresist verifies successful pattern transfer into the substrate. Stylus profilometer measurements were performed at three locations on each of two independently fabricated samples, as shown in Figure 10. The six location-level measurements yielded a mean etch depth of 626.5±6.4nm (mean ± SD). This value represents the etch depth in the fused-silica substrate and is distinct from the designed 1μm height of the TPL-fabricated photoresist mask.

To further evaluate the mechanical robustness of the transferred microstructures, the samples were continuously cleaned in an ultrasonic bath for 40 h. As shown in Figure 9d,h, although the photoresist layer is completely removed after prolonged cleaning, the transferred QR microstructures remain intact without observable structural damage or collapse. These results indicate that the QR microstructures are permanently transferred into the fused silica substrate and exhibit excellent resistance to prolonged mechanical agitation.

In addition to fabrication robustness, the storage capacity of the proposed architecture was estimated based on the demonstrated feature dimensions and QR configuration. With one physical bit represented by a 500nm×500nm module over a 127mm×127mm substrate, the theoretical upper-bound capacity without QR-encoding redundancy is

(1) $$C_{\mathrm{raw}}=\frac{127\;\mathrm{mm}\times 127\;\mathrm{mm}}{500\;\mathrm{nm}\times 500\;\mathrm{nm}}\times \frac{1}{8}\approx 8.06\;\mathrm{GB}.$$

For the projected single-layer storage configuration, QR Version 6 with error-correction level M was adopted, providing a maximum user-data payload of 106 bytes in byte mode. A Version 6 QR code consists of 41×41 modules, and including the standard four-module quiet zone on each side results in a 49×49-module storage unit. With a designed module width of 0.5μm, the physical footprint of each QR storage unit is 24.5×24.5μm^2^. Assuming a square-grid arrangement on a 127×127 mm^2^ fused-silica substrate, without additional spacing or edge margins, the number of complete QR units along each direction is

(2) $$N_{\mathrm{side}}=\left\lfloor \frac{127000}{49\times 0.5}\right\rfloor =5183.$$

The nominal projected user-data capacity is therefore calculated as

(3) $$C_{\mathrm{nominal}}=N_{\mathrm{side}}^{2}\times 106\approx 2.85\times 10^{9}\;\mathrm{bytes}=2.85\;\mathrm{GB}.$$

Thus, 8.06GB represents the theoretical physical upper bound, whereas 2.85GB represents the nominal effective user-data capacity under the adopted V6-M configuration, assuming successful fabrication of all QR storage units. Therefore, the estimated capacity should be regarded as a theoretical projection rather than an experimentally validated usable storage capacity.

### 3.5. Microscopic Readout and QR Reconstruction

#### 3.5.1. Readout and Reconstruction of Scan-Line QR Microstructures

**Figure 11:**
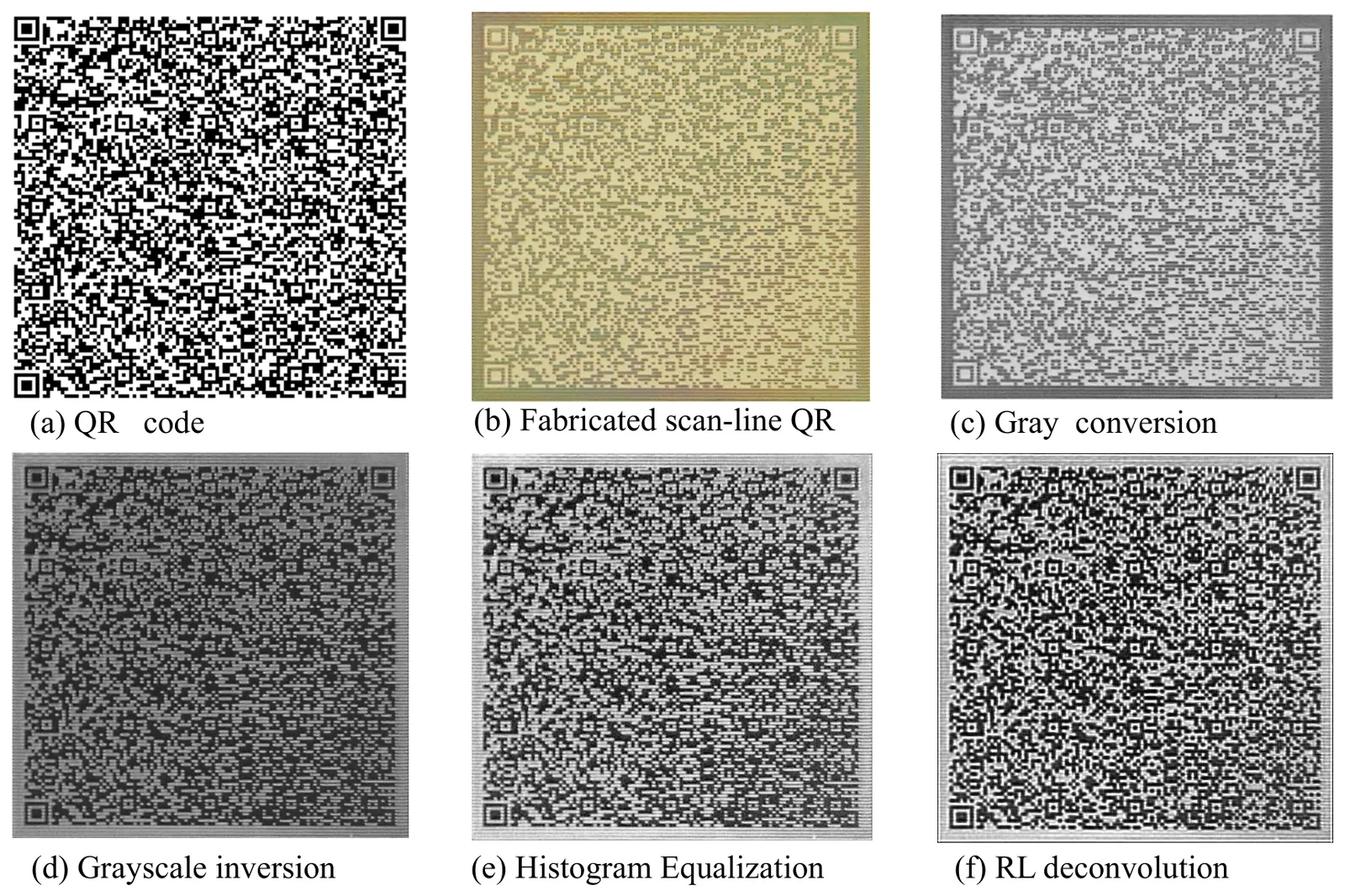
Image enhancement and restoration of scan-line QR microstructures. (**a**) QR code. (**b**) Raw microscopic image of a fabricated Version 23 scan-line QR microstructure. (**c**) Grayscale conversion. (**d**) Grayscale inversion. (**e**) Histogram equalization. (**f**) Richardson–Lucy (RL) deconvolution.

After microscopic imaging, the captured scan-line QR microstructures usually exhibit non-uniform background illumination, low contrast, and optical blur, which degrade the visibility of the embedded QR codes. To facilitate reliable decoding, a conventional image restoration pipeline is first applied, as illustrated in Figure 11. The captured microscopic image is sequentially processed by grayscale conversion, grayscale inversion, histogram equalization, and Richardson–Lucy deconvolution.

Grayscale conversion first transforms the captured color image into a single-channel intensity image for subsequent processing (Figure 11c). The grayscale image is then inverted to restore the original black–white polarity of the QR code. This operation reverses the grayscale inversion performed during QR preprocessing before applying the scan-line writing strategy, ensuring that the recovered scan-line image has the same polarity as the original QR code (Figure 11d). Histogram equalization is subsequently employed to redistribute image intensities and improve global contrast, making the scan-line structures more distinguishable (Figure 11e). Finally, Richardson–Lucy deconvolution compensates for optical blur introduced during microscopic imaging, thereby sharpening the structural boundaries of the fabricated microstructures (Figure 11f).

Figure 11 presents the intermediate results of each processing stage using a representative Version 23 scan-line QR microstructure. As the processing progresses, the background illumination is effectively suppressed, the contrast between the scan-line structures and the background is significantly enhanced, and the structural boundaries become progressively sharper. The final restored image clearly preserves the scan-line pattern and can be directly decoded by a standard QR decoder, demonstrating the effectiveness of the proposed image restoration pipeline. It should be noted that the digital image pixels in Figure 11 are distinct from the physical QR modules and the TPL polymerization voxels. The physical dimensions of the QR modules are defined by the designed fabrication geometry, whereas the dimensions of the TPL polymerization voxel depend on the optical focusing and exposure conditions. The microscope image pixels represent spatial sampling during optical readout and do not directly correspond to individual polymerization voxels.

#### 3.5.2. Readout and Reconstruction of QR Microstructures

Unlike scan-line QR microstructures, full-raster fabricated QR microstructures exhibit different imaging characteristics under microscopic observation. Owing to optical scattering and reduced intensity contrast inside the engraved regions, the interiors of QR modules become nearly indistinguishable from the background, leaving only their boundaries clearly visible. Consequently, the captured images exhibit contour-dominated appearances that cannot be reliably restored using conventional image enhancement methods.

To address this issue, the proposed hierarchy-aware contour filling algorithm is employed to reconstruct complete QR modules from the preserved contour information. After image deblurring and binarization, closed contours are extracted and organized into a parent–child hierarchy according to their nesting relationships. Starting from the outermost contour, the enclosed regions are recursively filled with alternating black and white colors according to the contour hierarchy, thereby reconstructing the original black–white topology of the QR code.

**Figure 12:**
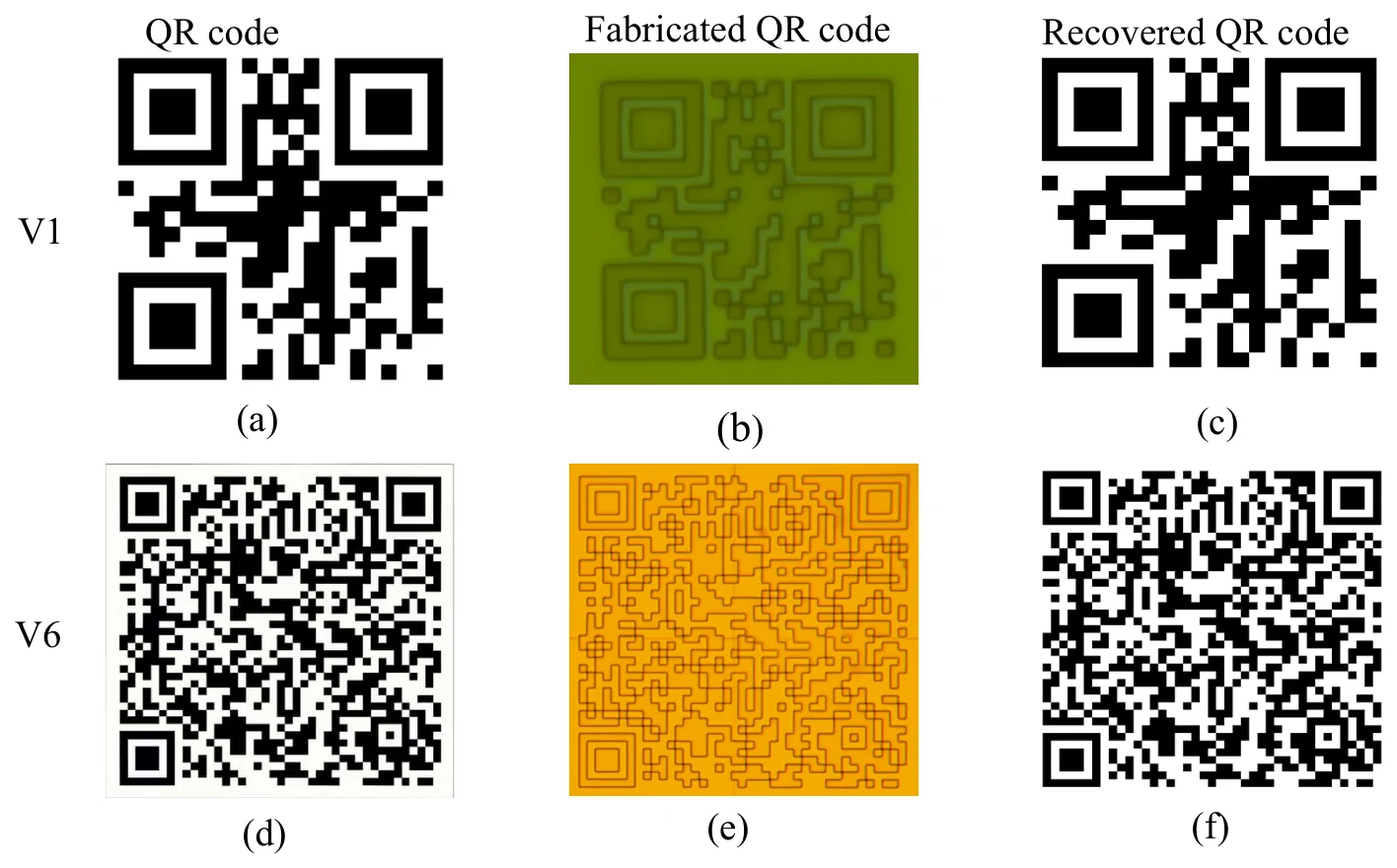
Recovery of conventionally fabricated QR microstructures using the proposed hierarchy-aware contour filling algorithm. (**a**) Original QR code (Version 1). (**b**) Microscope image of the fabricated QR microstructure. (**c**) Reconstructed QR code after image deblurring and hierarchy-aware contour filling. (**d**–**f**) Corresponding results for Version 6.

Representative reconstruction results are shown in Figure 12. Despite the severe degradation in the captured microscopic images, both Version 1 and 6 QR microstructures are successfully reconstructed into standard QR codes. These results demonstrate that the proposed hierarchy-aware contour filling algorithm effectively reconstructs standard QR codes from microscopic images, enabling reliable information retrieval using conventional optical microscopy.

Although Figure 12 presents Versions 1 and 6 as representative examples, the reconstruction algorithm was also examined on higher-version QR codes with more complex module distributions. Successful reconstruction and decoding were obtained for most of the examined higher-version patterns, including Version 30. However, increasing the QR version generally introduces more complex local module arrangements and deeper contour hierarchies. For a small number of patterns containing four-level nested contours, the recursive alternating filling may incorrectly assign the polarity of some local regions, causing a small number of module reconstruction errors. In the cases, the reconstruction errors remained within the error-correction capability of the QR codes and did not prevent successful decoding. To evaluate the readout performance, 200 successfully fabricated QR microstructures were subjected to the complete readout procedure, including microscopic imaging, image reconstruction, and standard QR decoding. All 200 successfully fabricated QR microstructures were correctly decoded after microscopic imaging and reconstruction, with the recovered payloads matching the originally encoded data. This result represents the decoding success rate conditional on successful fabrication and should be distinguished from the overall fabrication success rate.

### 3.6. Accelerated Thermal Aging and Lifetime Estimation

**Figure 13:**
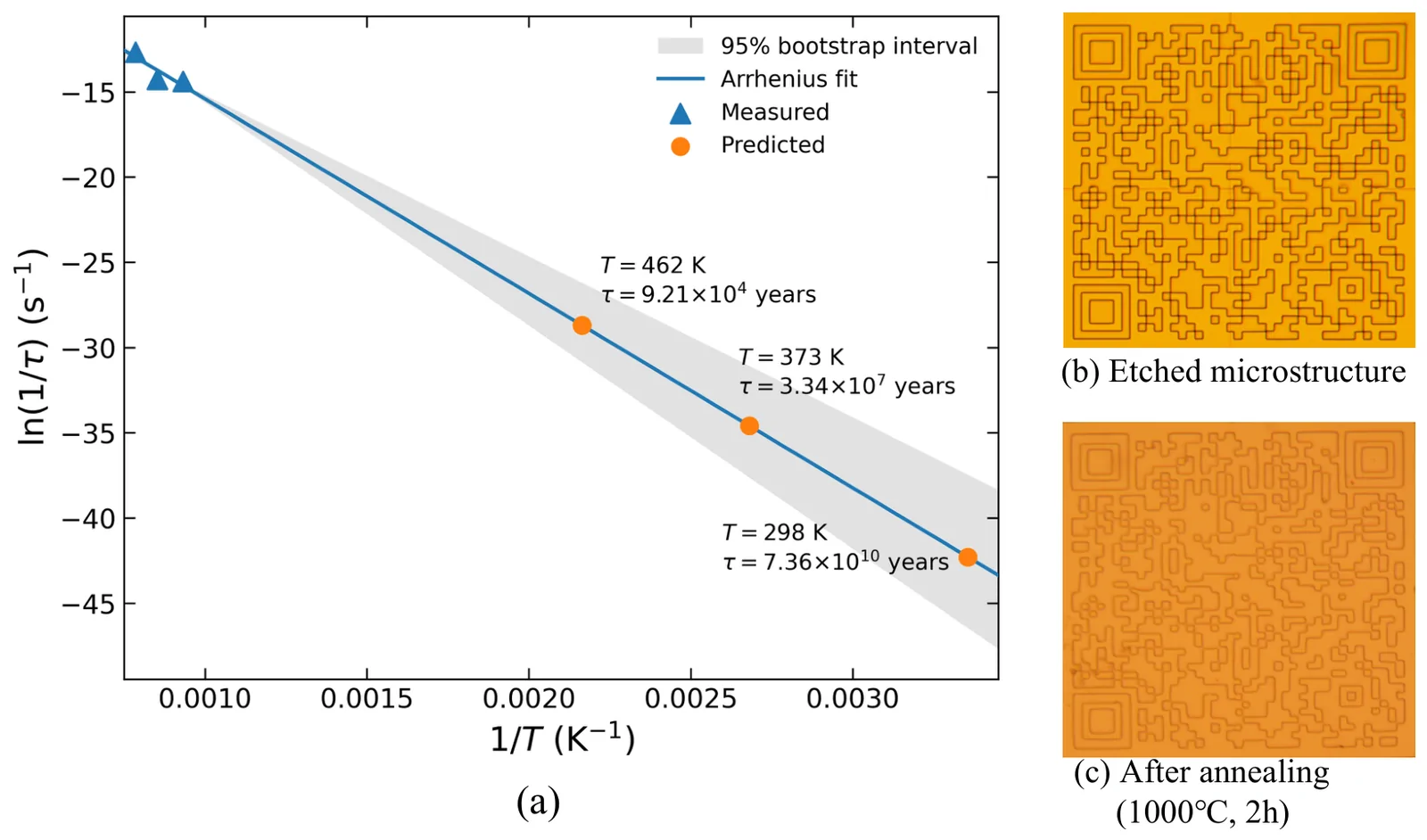
Accelerated thermal-aging evaluation of the RIE-transferred fused-silica QR microstructures. (**a**) Arrhenius analysis of the characteristic thermal-decay rate. The solid line represents the best-fit Arrhenius relation, and the shaded region denotes the 95% bootstrap interval obtained by propagating the uncertainty of the thermal-decay fitting through the Arrhenius analysis. (**b**) Optical microscope image of an as-etched QR microstructure before thermal aging. (**c**) Optical microscope image of the same QR microstructure after thermal aging at 1000 °C for 2 h.

To further evaluate the thermal stability of the RIE-transferred fused-silica QR microstructures, accelerated thermal-aging experiments were performed at various annealing temperatures ranging from 800 to 1000 °C. The microstructure height was measured at different annealing times and normalized to the initial value at each temperature. The QR microstructures remained clearly distinguishable and decodable after the thermal-aging treatments, while only gradual dimensional changes were observed as shown in Figure 13c.

Assuming that the thermally activated structural evolution follows an Arrhenius law: $\frac{1}{\tau }=A\exp\left(-\frac{E_{a}}{k_{B}T}\right)$, the measured decay rates were fitted according to $\ln\left(\frac{1}{\tau }\right)=\ln A-\frac{E_{a}}{k_{B}T}$, where *A* is the pre-exponential factor, $E_{a}$ is the apparent activation energy, and $k_{B}$ is the Boltzmann constant. As shown in Figure 13a, the high-temperature measurements were used to establish the Arrhenius relation and extrapolate the characteristic lifetime to lower temperatures. The fitted apparent activation energy is approximately 0.983eV. A parametric bootstrap analysis was further performed to propagate the uncertainty of the thermal-decay fitting through the Arrhenius extrapolation. The 95% bootstrap interval for $E_{a}$ is 0.815–1.128eV, and the corresponding uncertainty in the Arrhenius relation is represented by the shaded region in Figure 13a. The best-fit characteristic-lifetime estimates are $9.21\times 10^{4}$, $3.34\times 10^{7}$, and $7.36\times 10^{10}$ years at 462, 373, and 298 K, with corresponding 95% bootstrap intervals of $7.62\times 10^{3}$–$8.02\times 10^{5}$, $9.97\times 10^{5}$–$6.94\times 10^{8}$, and $5.90\times 10^{8}$–$4.75\times 10^{12}$ years, respectively.

These accelerated-aging results demonstrate the high thermal stability of the etched fused-silica surface-relief microstructures. However, the increasing uncertainty toward lower temperatures reflects the large extrapolation beyond the experimental temperature range. The estimated lifetimes should therefore be interpreted as model-dependent thermal stability estimates rather than precise predictions of archival lifetime. Other long-term degradation mechanisms, including humidity, contamination, chemical exposure, and mechanical damage, are not included in the present model and require further investigation.

**Environmental Stability and Limitations.** Although fused silica exhibits excellent intrinsic thermal and chemical stability, the environmental durability of surface-relief microstructures must be distinguished from that of volumetric structures embedded within the glass. In volumetric storage, the surrounding glass provides additional protection against external environmental influences, whereas surface-relief QR microstructures are directly exposed to the environment. In this study, accelerated thermal-aging experiments demonstrated the thermal stability of the RIE-transferred QR microstructures under the investigated conditions, while prolonged ultrasonic cleaning provided additional evidence of structural integrity under mechanical agitation. However, these results do not establish long-term resistance to other environmental hazards, including humidity, surface contamination, chemical exposure, and mechanical abrasion, which may alter the microstructure geometry or reduce optical readout reliability. Therefore, the Arrhenius-based lifetime estimates should be interpreted as predictions of thermally activated structural stability rather than comprehensive archival lifetime guarantees. For practical long-term storage applications, protective packaging may help reduce environmental exposure, while systematic environmental reliability testing will be necessary to evaluate the effects of humidity, chemical exposure, contamination, and mechanical damage on QR structure integrity and decoding performance.

### 3.7. Comparison with Existing Fused-Silica Storage Approaches

In this subsection, we compare our system with two representative fused-silica storage approaches: the Project Silica system (PS) [19] and the Driveless Read system (DR) [22]. Table 2 provides a parameter-wise comparison. The main similarities and differences are summarized as follows:

1. All three systems employ femtosecond-laser-based microstructuring of fused silica for long-term digital storage.
2. PS and DR adopt multilayer volumetric storage architectures implemented using custom femtosecond laser writing systems. In contrast, our system uses a commercial TPL system and a single-layer surface-relief architecture.
3. Our system has an estimated total areal storage density of 0.177 MB/mm^2^, approximately 2.8 times that of the DR system when all four recording layers of DR are included in the comparison. PS achieves a significantly higher storage density by utilizing more sophisticated and specialized hardware.
4. Error correction coding (ECC) is implemented in both PS and our system, whereas no ECC implementation was reported for DR.
5. We explicitly evaluate reliability by our measurement of the fabrication success rate. A success rate of 94.5% is obtained, which we consider practically viable. Directly comparable fabrication-yield statistics based on the same structural-integrity criterion are not available for the reference systems considered in Table 2.

In summary, compared with PS and DR, our design offers simplicity while maintaining a storage density competitive with that of DR and an acceptable fabrication success rate. The proposed approach therefore represents an alternative fused-silica storage architecture that prioritizes conventional optical readout, while acknowledging the trade-offs in storage density, fabrication throughput, and system scalability.

## 4. Conclusions

In this work, we demonstrated a direct-readout optical archival storage framework based on permanent QR microstructures in fused silica. Unlike birefringence-based storage systems and volumetric dot-based direct-readout approaches, the proposed framework employs TPL-assisted patterning and RIE transfer to form surface-relief QR microstructures and focuses on improving fabrication efficiency and microscopic reconstruction. By combining QR encoding, two-photon lithography, reactive ion etching, and microscopic image reconstruction, the proposed approach establishes a practical workflow for accessible long-term optical archival storage.

Experimental results demonstrate the effectiveness of the proposed framework from fabrication to readout. The scan-line QR writing strategy reduces the TPL writing time by approximately 80% while preserving the encoded information. Reliable QR microstructures with module widths down to 500 nm were successfully transferred into fused silica and remained structurally intact after 40 h of ultrasonic cleaning, confirming excellent mechanical robustness. For QR Version 6 with M-level error correction, the scan-line writing strategy achieved a fabrication success rate of 94.5% using 1 µm modules. Separately, assuming 500 nm modules and the specified QR packing configuration, the estimated geometric storage capacity is approximately 2.85 GB per disc. This capacity remains a theoretical projection rather than an experimentally demonstrated full-disc storage capacity.

The proposed framework is primarily intended for write-once, read-many (WORM) archival storage. Although TPL, RIE, and cleaning introduce additional complexity during the initial data-writing process, these steps are performed only once and can be carried out in a dedicated fabrication facility. Once the QR microstructures are permanently transferred into fused silica, subsequent data retrieval requires only conventional optical microscopy and QR reconstruction, without additional lithography or etching equipment. Therefore, the main practical advantage of the proposed framework lies in simplifying repeated data access rather than the initial fabrication process. Nevertheless, the multi-step fabrication procedure remains a challenge for large-scale commercialization, and future work will focus on improving fabrication throughput and process integration.

The proposed framework provides a practical route toward long-term and easily accessible optical archival storage. Future research can further extend the proposed framework in several directions. One direction is to develop fabrication-oriented encoding strategies for higher storage density and improved fabrication reliability. Another is to further optimize the scan-line writing strategy to increase TPL writing throughput.

## Figures and Tables

**Table 1:** Selected RIE parameters for QR microstructure transfer.

Parameter	Value
${\mathrm{CF}}_{4}$ flow rate	30 SCCM
Ar flow rate	10 SCCM
RF power	170 W
Process time	15 min

**Table 2:** Comparison of representative fused-silica optical data-storage approaches.

Item	Project Silica [19]	Driveless Read System [22]	This Work
Architecture	301-layer volumetric birefringent-voxel	4-layer volumetric binary-dot	Single-layer surface relief QR code
Writing system	Custom-built fs-laser system	Custom-built fs-laser system	Commercial TPL system
ECC	LDPC + erasure coding	No ECC reported	RS code, level M
Fabrication reliability	Not reported	Not reported	94.5%
Readout system	Polarization-resolved microscopy	Low-magnification optical microscope	Conventional bright-field optical microscope
Decoding	CNN-based symbol inference + LDPC	Image processing + binary-dot detection	Image processing + standard QR decoder
Readout performance	Full user-data recovery using FEC	BER∼$10^{-3}$ without ECC	200/200 tested QR correctly decoded
Capacity	$336\;\mathrm{MB}/{\mathrm{mm}}^{2}$ *	$0.062\;\mathrm{MB}/{\mathrm{mm}}^{2}$ *	$0.177\;\mathrm{MB}/{\mathrm{mm}}^{2}$ *

ECC: Error Correction Coding; LDPC: Low-Density Parity-Check; RS: Reed–Solomon; Values marked with * are derived from unit conversions.

## Data Availability

Data underlying the results presented in this paper are not publicly available at this time but may be obtained from the authors upon reasonable request.
